# Quantification of *cki-1* promoter activity during PHso1-to-PHD remodeling in *Caenorhabditis elegans*

**DOI:** 10.17912/micropub.biology.002243

**Published:** 2026-07-22

**Authors:** Nathazsha Gande, Nicholas Palmisano, Aram Stump

**Affiliations:** 1 Division of Biosciences, University College London, London WC1E 6BT, United Kingdom; 2 Department of Biology, Adelphi University, Garden City, NY, United States

## Abstract

Cyclin-dependent kinase inhibitor ( *cki-1* ) is associated with cell cycle arrest and cellular quiescence. In *Caenorhabditis elegans* , glia-to-neuron transdifferentiation of the phasmid socket 1 (PHso1) glia into the phasmid D (PHD) neuron has been previously described across larval stages. Here, we report the post-hatching timeline of PHso1-to-PHD remodeling within L4 substages, including the loss of glial socket morphology and the acquisition of neuronal features well into adulthood. We find that *cki-1* expression decreases across L4 substages between 40-50 hours post-hatching in males, while *cki-1* remains robustly expressed in hermaphrodite PHso1 cells. Across L3, L4, and adulthood, PHso1 cell proportions change consistently.

**Figure 1. Sex-specific temporal downregulation of cki-1 during PHso1-to-PHD transdifferentiation:** **A)** *cki-1 ^prom^ ::GFP* expression intensity of PHso1-to-PHD remodelling in males (solid circles) and unchanged PHso1 glia in hermaphrodites (dashed circles). **B)** Mean GFP intensity in PHso1/PHD cells of male and hermaphrodite (n=8) at Early L4 (40–42 hr), Mid-L4 (46 hr), Late L4 (48 hr), and Adult (50 hr) developmental stages. A two-way ANOVA and Tukey's post-hoc comparisons show significant differences between timepoints (p < 0.05). Solid gray lines represent hermaphrodite PHso1 glial cells, while black lines represent male PHso1/PHD cells. Error bars represent ± S.D. from the mean. **C)** Remodeling of PHso1-to-PHD labeled in red with *lin-48 ^prom^ ::tdTomato* in males and hermaphrodites. Arrowheads indicate the progressive extension of the nascent axonal process projecting anteriorly toward the pre-anal ganglion (PAG), while asterisks indicate the posterior dendritic process. Exposure was optimized to visualize these neuronal processes, resulting in overexposure of the bright signal on the left side of the image. **D)** Percentage of PHso1 cells per side at the L3, L4, and adult stages in males. Black indicates zero PHso1 cells and gray represents more than one PHso1 glia per side. **E)** Percentage of PHso1 glia in hermaphrodites from L3 stage to adulthood.

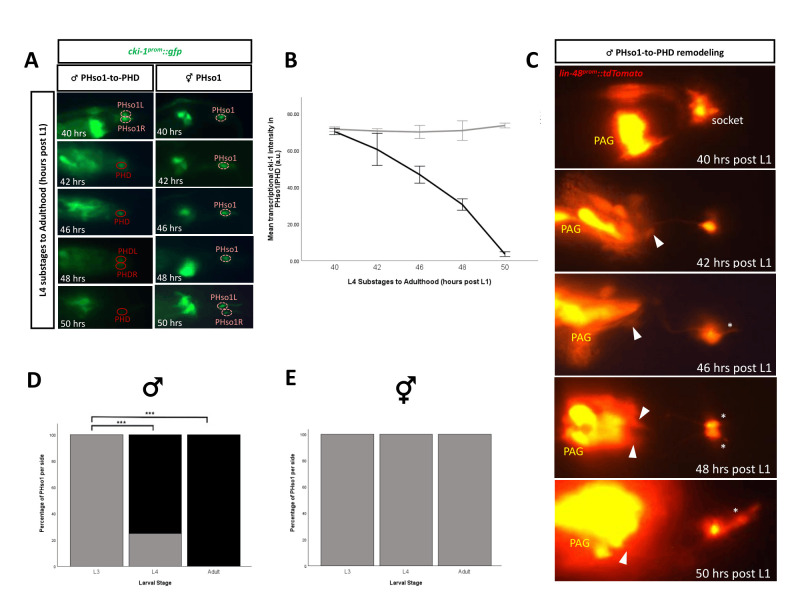



## Description

Proper development of a multicellular organism requires the precise coordination of cell cycle control and differentiation (Hindley and Philpott, 2012; Hong et al., 1998). In *Caenorhabditis elegans* , sexually dimorphic sensory-motor circuits emerge from processes such as direct glia-to-neuron transdifferentiation during development (Molina-Garcia and Lloret-Fernandez et al., 2020). In the tail, the bilateral phasmid sensilla contains two phasmid socket glia (PHso1 and PHso2) (Sulston et al., 1980). Sex-specific remodeling of the sex-shared PHso1 undergoes direct (without cell division) transdifferentiation into the phasmid D (PHD) neuron in male *C. elegans ,* whereas glial identity remains unchanged in hermaphrodites through to adulthood (Molina-Garcia and Lloret-Fernandez et al., 2020; Sammut, 2022).

Critically, cell cycle regulators such as Cyclin-Dependent Kinase Inhibitors (CKIs) regulate the transition between cell cycle phases and are associated with neuronal fate specification through Cyclin-Cyclin Dependent Kinase (CDK) complex formation (Sammut, 2022), subcellular localization (Kreis et al., 2019), and proteasomal degradation (Al Bitar & Gali-Muhtasib, 2019).

In *C. elegans* , *cki-1* is a member of the highly conserved CIP/KIP family (Buck et al., 2009). For instance, in the embryonic worm, *cki-1* is required for cell cycle arrest and its knockdown results in excess cell divisions and inhibits the activities of Cyclin-CDK complexes that regulate the G1/S phase, resulting in G1 cell cycle arrest (Fukuyama et al., 2003; Sherr and Roberts, 1995). Previous work demonstrated that *cki-1* is expressed strongly in sex-shared PHso1 glia at the L3 stage and no longer detected in male young adults, although it is consistently expressed throughout life in hermaphrodite PHso1 (Sammut, 2022).

Here, we used the *cki-1* transcriptional reporter transgene [ *cki-1 prom::gfp* + *dpy-20* (+)] (Hong et al., 1998) together with a *lin-48* transcriptional reporter transgene [ *lin-48 prom::tdTomato* ] as a PHso1/PHD marker to quantify *cki-1* promoter activity to characterize the hours post-hatching timeline of PHso1-to-PHD remodeling across L4 substages in males. Additionally, we compared *cki-1* promoter activity dynamics with hermaphrodite PHso1 cells.

In the L4 substages of males (40 to 50 hours post L1), PHso1 cells are still present with a strong *cki-1 ^prom^ ::gfp* expression at 40 hours post L1. At this stage the PHso1 cells have glial characteristics and do not show any clear neuronal features indicated by the absence of axonal and dendritic processes. Given that there are no large *cki-1* intensity differences between 40 and 42 hours post L1 (mean difference [MD] = 9.75, p = 0.129), these time points may indicate that PHso1 glia are still retracting their socket processes and starting to undergo neurogenesis (Fig 1A and B). Morphological transformations are notable by 42 hours post L1, where early neuronal properties are seen (a nascent axon projecting anteriorly) from the soma of the PHso1-derived neuron (Fig 1C). Using conventional wide-field fluorescence microscopy, Figure 1C shows the neuronal processes highlighted by the arrowheads. From 42 hours to 50 hours post L1, *cki-1 ^prom^ ::gfp* intensity is shown to downregulate and the cell identified as a PHD neuron(Fig 1B and C). At 46 hours post L1, the nascent axon extension remains incomplete and does not fully integrate with its postsynaptic targets within the pre-anal ganglion (PAG). By the late L4 stage (48 hours post L1), full extension of the axon is complete with axonal growth cones reaching the postsynaptic interneurons located in the PAG (Fig 1C). Dendritic elongation was also visible which is characteristic of PHD neuron maturation (Fig 1C). By adulthood, (50 hours post L1 onward), adult males have PHD neurons and mature features of all nine sensory rays that are fully formed, where *cki-1 ^prom^ ::gfp* was evidently absent (Fig 1A and B, *Extended data* ). Overall, in males, *cki-1 ^prom^ ::gfp* expression had significantly higher GFP intensity at earlier L4 timepoints (40 and 42 hours post L1) than later L4 timepoints (46, 48, and 50 hours post L1). The downregulation in *cki-1 ^prom^ ::gfp* intensity was most pronounced among 40 and 50 hours post L1 (Fig 1B) (MD = 66.75, p < 0.001). Tukey's post-hoc analyses demonstrated statistically significant pairwise differences between 40 and 50 hours (MD = 66.75, p < 0.001), 46 and 48 hours (MD = 16.25, p = 0.005), and 46 and 50 hours (MD = 43.25, p < 0.001), which is a distinct change of transcriptional *cki-1* dynamics coinciding with the loss of glial socket morphology and acquisition of neuronal features at L4 substages post L1.

In hermaphrodites, PHso1 glia were identified at all L4 substages from 40-50 hours post L1, and *cki-1* is robustly expressed throughout (Fig 1A and B). Pairwise comparisons revealed no significant differences between L4 substages in hermaphrodites (MD ranging from -3.5 to 3.5, all *p > 0.05* ) indicating that *cki-1* expression remains unchanged within the L4 stage. Given that PHso1 cells in hermaphrodites do not undergo transdifferentiation, this stability in *cki-1* expression aligns with their quiescent state.

We next asked whether the number of PHso1 glia per side would drop across developmental stages such as L3, L4, and adulthood to determine whether their fate was constant across sexes. In L3, 100% of males and hermaphrodites kept PHso1 glia present in the phasmid sensillum, which do not transdifferentiate (Fig 1D and E). Hermaphrodites maintain their glial identity from L3 through adulthood with evidence of a persistent socket process morphology and *cki-1 ^prom^ ::gfp* expression remained apparent throughout L3, L4, and adulthood (see *Extended data* ).

Nonetheless, in L4, 25% of males displayed at least one PHso1 glia on each side that corresponded with maintaining glial identity such as the presence of a socket process. The remaining 75% of males exhibited a loss of PHso1 glia on at least one side, which correlated with the emergence of neuronal characteristics (e.g., nascent axon extension and dendritic elongation), where *cki-1* transcription is repressed (Fig 1C and D). It was confirmed that by adulthood, all males had completely lost PHso1 glia, which transitioned into PHD neurons (Fig 1D).

## Methods

*Age Synchronization at L1-arrest*

Gravid adults expressing *maIs113 [ cki-1 ::gfp+dpy-20(+)]* and *drpIs3 [ lin-48 ::tdTomato]; him-5 ( e1490 )V* were collected from NGM plates by washing with M9 buffer and pelleted by centrifugation at 400 x g (1500 rpm) for 2 minutes. The pellet was washed 1-3 times until M9 buffer was clear of bacteria. To synchronize worms, gravid adults were treated with an alkaline hypochlorite solution (3.5ml of H _2_ O, 0.5ml (5M) NaOH, and 1ml of 3% sodium hypochlorite) and vortexed for 6 minutes to degrade adult tissue, with monitoring under a dissecting microscope. When no traces of adult bodies were present, the reaction was stopped by adding 6mL of M9 buffer to the alkaline hypochlorite solution containing specimens. The sample was centrifuged at 400xg for 1 minute to pellet *C. elegans* embryos and to allow for removal of residual bleach by decanting. The pellet was washed three more times with M9 buffer and then resuspended in 1 ml of M9 buffer. The eggs were incubated at 20°C in M9 buffer to allow hatching L1s to synchronously arrest in development.

*Image Acquisition and Quantification*

Differential Interference Contrast (DIC) and fluorescence images were acquired using a Zeiss Axioskop 50 microscope equipped with a Lumenera INFINITY3-6URC camera and INFINITY CAPTURE software. This conventional wide-field imaging setup does not provide the optical sectioning possible with confocal or Z-stack imaging. Animals were mounted on 2% agarose pads and immobilized with 10 uL of polystyrene latex beads (0.1 um mean particle size) in 10uL of M9 buffer on a standard microscope slide. Imaging was carried out using a Plan-NEOFLUAR 40x (NA 0.75) objective lens with immersion oil. 

Fluorescence imaging of males and hermaphrodites carrying *cki-1 ^prom^ ::gfp* and *lin-48 ^prom^ ::tdTomato* reporter transgenes were done to visualize their expression. In order to standardize samples, exposure time and gain were kept constant during the imaging process. Exposure settings were selected to maximize visualization of the relatively weak neuronal processes of PHso1/PHD, resulting in partial saturation of brighter fluorescent structures in some images. Excitation of GFP fluorescence used a 480 nm excitation filter, and emission was between 535 nm to optimize signal detection. The extent of tail tip development and retraction relative to hermaphrodite vulva morphogenesis was used to indicate the larval stages and subsequent L4 substages of the nematodes (Kiontke at al., 2024). To quantify fluorescence intensity, images were processed using the National Institute of Health-funded software, ImageJ. Initial processing involved splitting the RGB color channels to focus on the green channel to quantify the mean gray value (MGV) intensity. In each image, a region of interest (ROI) in the cell body of each cell was drawn using the polygon tool to cover the area that is selected. For each larval stage (L3, L4, and adult), or within L4 substages, MGV was quantified in the cell bodies of PHso1 and PHD cells. Background fluorescence was subtracted from each measurement.

*Statistical analysis*

Statistical analyses were performed to determine differences in *cki-1 ^prom^ ::gfp* expression between developmental stages, cell types, and sexes. Data were analyzed in SPSS (version 29.0) and Graphpad Prism. All statistical tests were carried out using a significance threshold of p < 0.05. A two-way analysis of variance (ANOVA) was conducted to examine differences in transcriptional *cki-1 ^prom^ ::gfp* expression of between the male and hermaphrodites across L4 developmental time points (hours post L1). The two-way ANOVA showed significance for L4 developmental timepoints between the sexes. This was followed by Tukey's Honest Significant Difference (HSD) post-hoc test within each sex. A Fisher's exact test for the 2×2 contingency table was used to assess sex differences in the proportion of PHso1 glia per side from L3 to adulthood in both males and hermaphrodites.

## Reagents

**Table:** 

Reagent type (species) or resource	Designation	Source or reference	Genotype
Strain background ( *E. coli* )	*OP50*	Caenorhabditis Genetics Centre	
Genetic reagent ( *C. elegans* )	*CHL142 *	Dr. Richard J. Poole laboratory	*maIs113 [ cki-1 ::gfp+dpy-20(+)]; drpIs3 [ lin-48 ::tdTomato]; him-5 ( e1490 )V *

## Data Availability

Description: DIC, tdTomato, and GFP images in L3, L4, and adult stages in males and hermaphrodites. Fluorescence images showing the expression of the quiescence marker, cki-1prom::GFP (green), and PHso1/PHD marker, lin-48prom::tdTomato (red). In hermaphrodites, PHso1 cells (pink dashed circles) at L3, L4, and adulthood maintains glial identity. Male PHso1 cells transitioning into PHD neurons (red circles) during the L4 stage show that cki-1 expression decreases in the cell body of developing neurons. Resource Type: Dataset. DOI: https://doi.org/10.22002/e5405-sww36
